# Age-related differences in clinical characteristics of Kawasaki disease

**DOI:** 10.1590/1414-431X202010281

**Published:** 2021-01-15

**Authors:** Yu Peng, Xiaohui Liu, Zhao Duan, Sufen Cai, Junkai Duan, Yulan Zhou

**Affiliations:** 1 Jiangxi Province Children's Hospital, Department of Rheumatology, NanchangJiangxi China Department of Rheumatology, Jiangxi Province Children's Hospital, Nanchang, Jiangxi, China; 2 Jiangxi Province Children's Hospital, Department of Cardiology, NanchangJiangxi China Department of Cardiology, Jiangxi Province Children's Hospital, Nanchang, Jiangxi, China; 3 The First Affiliated Hospital of Nanchang University, Department of Hematology, NanchangJiangxi China Department of Hematology, The First Affiliated Hospital of Nanchang University, Nanchang, Jiangxi, China

**Keywords:** Kawasaki disease, Infants, Coronary artery lesion, Clinical characteristics

## Abstract

This study aimed to examine and summarize clinical characteristics of Kawasaki disease (KD) at different ages to further strengthen clinicians understanding of children with KD, improving the level of diagnosis, and reducing coronary artery complications of KD. A total of 398 patients with KD who were diagnosed between January 2016 and December 2017 were reviewed retrospectively. These participants were allocated into three groups according to age: group A (<1 year, n=62), group B (≥1 and <5 years, n=286), and group C (≥5 years, n=50). Clinical manifestations, laboratory results, and echocardiographic findings were compared among the groups. Most (71.86%) patients with KD were aged 1-5 years. The prevalence of cervical lymphadenopathy was lowest in group A. The duration of fever before admission was longest in group A. The rate of cervical lymphadenopathy and laboratory data were different among the groups. Group A had higher frequencies of gastrointestinal involvement, neurological symptoms, and redness at the Bacillus Calmette-Guerin inoculation site than the other groups. Infants aged <1 year with KD often have a longer duration of fever before admission, a lower prevalence of cervical lymphadenopathy, and a higher prevalence of gastrointestinal and neurological symptoms.

## Introduction

Kawasaki disease (KD) is an acute, self-limited vasculitis with a worldwide occurrence in children of different ethnic origins. Its prevalence is significantly higher in North-East Asian countries (notably Japan and China) than in non-Asian ethnicities like the United States (1,2). Due to different meteorological factors, infectious agents, and improved economic conditions, previous studies have revealed changes in epidemiology of KD over time (3). The incidence rate increases yearly. Recently, several cases of Kawasaki-like disease have been reported in various locations that coincide with the Sars-CoV-2 pandemic, making this disease one of the hot topics (4,5). Most cases of KD occur between the ages of 1 and 5 years (1,6), with fewer cases in children younger than 1 year or older than 5 years. These younger and older patients are more likely to have the incomplete type of KD. Therefore, diagnosis and treatment of KD could be delayed. Additionally, patients with delayed treatment have a higher risk of coronary artery lesions (CALs), which is the most serious complication of KD.

To increase clinicians understanding of children with KD, improving the level of diagnosis, and reducing the coronary artery complications of KD, we investigated the clinical characteristics of patients with KD at different ages to determine age-related differences in the course of this disease.

## Material and Methods

### Patients

We retrospectively reviewed the medical records of patients who had KD on admission at the Department of Rheumatology, Jiangxi Province Children's Hospital between January 2016 and December 2017. Patients who had insufficient clinical or laboratory data were excluded. Patients who fulfilled the diagnostic criteria of complete or incomplete KD according to the American Heart Association diagnostic guidelines (7) were enrolled in this study. The data were categorized into the following three groups by age at diagnosis: group A (<1 year, n=62), group B (≥1 year and <5 years, n=286), and group C (≥5 years, n=50). The study was approved by the Medical Ethics Committee of Jiangxi Province Children's Hospital (JXSETYY-YXKY-20180031). Written informed consent was not required because of the retrospective nature of the study, and all identifying information of the enrolled patients was protected.

### Definitions

CALs were defined as an internal lumen diameter ≥3 mm in children aged <5 years or ≥4 mm in children aged ≥5 years (8). Intravenous immunoglobulin (IVIG) resistance was defined as recurrent or persistent fever for at least 36 hours after the end of IVIG administration (7). Gastrointestinal disorders included abdominal pain, hepatitis, diarrhea, vomiting, jaundice, gallbladder hydrops, and pancreatitis. Respiratory disorders included peribronchial and interstitial infiltrates on chest radiography. Genitourinary disorders included urethritis, hydrocele, and phimosis. Neurological disorders included aseptic meningitis, extreme irritability, and transient peripheral facial nerve palsy. Musculoskeletal disorders included arthralgia and arthritis. Redness at the Bacillus Calmette-Guerin (BCG) inoculation site, which was recorded precisely before and during hospitalization, was defined as any redness, induration, or crust formation.

### Data collection

Data on demographic, clinical, laboratory, and echocardiographic characteristics were recorded. Demographic and clinical characteristics included age, sex, KD-associated manifestations, and clinical outcomes. Laboratory tests were performed on the first day of hospitalization. Two-dimensional echocardiography was used to assess cardiovascular complications, and this was performed at the time of diagnosis, several times a week during hospitalization, and 2 months after diagnosis.

### Statistical analysis

Numerical data are reported as means±SD or median with interquartile range. Categorical variables are reported as numbers and percentages. We used one-way analysis of variance to analyze differences between groups based on numerical variables that showed a normal distribution. For analysis of variables not showing a normal distribution, we used the Kruskal-Wallis test. For analysis of categorical variables, we used the chi-squared test or Fisher's exact test. For variables that were significantly different among the three groups, pairwise comparisons were performed using the Bonferroni *post hoc* test. Statistical analyses were performed using SPSS version 22.0 for Windows (IBM, USA). A two-sided P<0.05 was considered to be statistically significant.

## Results

### Clinical characteristics among patients with KD with different ages

Three hundred ninety-eight patients with KD with an age range of 2 months to 12 years were enrolled in this study. Baseline characteristics are summarized in Table 1. There was a significant difference in age at diagnosis of KD among the three groups (P<0.001). There were 258 (64.82%) males in this cohort, with no significant difference in sex distribution among the different age groups. The prevalence of incomplete type of KD was significantly different among the groups (P<0.001). Group B had a relatively lower proportion of the incomplete type than group A, but there was no significant difference between groups A and C and groups B and C. The duration of fever before admission was significantly longer in group A than in the other age groups (P=0.034). Except for fever, the most frequent symptom was bulbar conjunctival injection (339 cases, 85.2%), followed by oral changes (326 cases, 81.9%), rash (244 cases, 61.3%), cervical lymphadenopathy (239 cases, 60.1%), and changes in the extremities (168 cases, 42.2%). The frequency of cervical lymphadenopathy was lowest in group A, with a significant difference among the groups (P<0.001). There were no significant differences in the frequency of rash, bulbar conjunctival injection, oral changes, and changes in the extremities among the different age groups.

**Table 1:** Clinical parameters in the different Kawasaki disease (KD) age groups.

Variable	Group A	Group B	Group C	F or χ^2^ value	P value
Number of patients	62	286	50		
Age at KD diagnosis (months)	8.48±1.12	24.15±14.67*	86.82±23.75*,**	173.67	<0.001^#^
Male, n (%)	40 (64.5%)	186 (65.0%)	32 (64.0%)	0.023	0.989
Incomplete KD, n (%)	28 (45.2%)	66 (23.1%)*	18 (36.0%)	14.036	<0.001^#^
Fever duration, days (mean±SD)	7.4±2.4	6.9±1.9*	7.1±2.1*	6.235	0.034^#^
Rash, n (%)	34 (54.8%)	184 (64.3%)	26 (52.0%)	4.025	0.134
Cervical lymphadenopathy, n (%)	18 (29.0%)	183 (64.0%)*	32 (64.0%)*	26.352	<0.001^#^
Conjunctivitis, n (%)	56 (90.3%)	242 (84.6%)	41 (82.0%)	1.771	0.412
Oral changes, n (%)	54 (87.1%)	234 (81.8%)	38 (76.0%)	2.306	0.316
Extremity changes, n (%)	22 (35.5%)	130 (45.5%)	16 (32.0%)	4.521	0.104
IVIG-resistant, n (%)	15 (24.2%)	52 (18.2%)	10 (20.0%)	1.196	0.550

Group A:<1 year; group B: ≥1 and <5 years; group C: ≥5 years; IVIG: intravenous immunoglobulin. ^#^P<0.05 among Groups A, B, and C; *P<0.05 compared with Group A; **P<0.05 compared with Group B. The chi-squared test, Fisher's (F) exact test, or one-way analysis of variance were used for statistical analyses.

Except for the classic clinical features, multiple other organs and tissues were inflamed during the acute illness and caused clinical symptoms. We analyzed these clinical symptoms in the different age groups. As shown in Table 2, 55 (13.8%) patients had gastrointestinal involvement, 69 (17.3%) had respiratory involvement, 33 (8.3%) had genitourinary involvement, 32 (8.0%) had neurological involvement, 43 (10.8%) had musculoskeletal involvement, and 45 (11.3%) had redness at the BCG inoculation site. There was a significant difference in gastrointestinal involvement among the groups (P=0.004), with group A having a higher frequency than group B, but with no significant difference between groups A and C and between groups B and C. The rates of neurological involvement and redness at the BCG inoculation site were significantly higher in group A than in groups B and C (P=0.003 and P<0.001, respectively). However, the frequency of musculoskeletal disorders in group A was significantly lower than that in groups B and C (P=0.043). There were no significant differences in the frequencies of respiratory disorders and genitourinary disorders among the different age groups. We further analyzed the number of patients with each gastrointestinal or neurological presentation in the different age groups. We found that the frequency of diarrhea and vomiting in group A was significantly higher than that in group B (P=0.006 and P=0.011, respectively), but there were no significant differences between groups A and C or between groups B and C. The frequency of extreme irritability was highest in group A (P<0.001). There were no differences in the frequencies of other gastrointestinal or neurological presentations among the different age groups.

**Table 2:** Other clinical findings in the different Kawasaki disease age groups.

Variable	Group A	Group B	Group C	χ^2^ value	P value
Gastrointestinal, n (%)	16 (25.8%)	30 (10.5%)*	9 (18.0%)	10.877	0.004^#^
Abdominal pain, n (%)	0 (0%)	15 (5.2%)	3 (6%)	3.941	0.147
Hepatitis, n (%)	0 (0%)	0 (0%)	1 (2%)	4.742	0.126
Diarrhea, n (%)	10 (16.1%)	13 (4.5%)*	2 (4%)	9.635	0.006^#^
Vomiting, n (%)	8 (12.9%)	10 (3.5%)*	1 (2%)	8.430	0.011^#^
Jaundice, n (%)	2 (3.2%)	1 (0.3%)	1 (2%)	5.116	0.069
Gallbladder hydrops, n (%)	1 (1.6%)	2 (0.7%)	2 (4%)	3.928	0.091
Pancreatitis, n (%)	0 (0%)	2 (0.7%)	1 (2%)	1.672	0.338
Respiratory, n (%)	8 (12.9%)	54 (18.9%)	7 (14.0%)	1.715	0.424
Genitourinary, n (%)	7 (11.3%)	21 (7.1%)	5 (10.0%)	1.264	0.532
Neurological, n (%)	12 (19.4%)	18 (6.3%)*	2 (4.0%)*	10.525	0.003^#^
Aseptic meningitis, n (%)	5 (8.1%)	14 (4.9%)	2 (4%)	1.279	0.550
Extreme irritability, n (%)	10 (16.1%)	9 (3.1%)*	0 (0%)*	15.978	<0.001^#^
Transient peripheral facial nerve palsy, n (%)	0 (0%)	3 (1%)	0 (0%)	0.395	1.000
Musculoskeletal, n (%)	1 (1.6%)	35 (12.2%)*	7 (14.0%)*	6.309	0.043^#^
Redness at the BCG inoculation site, n (%)	27 (43.5%)	18 (6.3%)*	0 (0.0%)*	77.811	<0.001^#^

Group A: <1 year; group B: ≥1 and <5 years; group C: ≥5 years. ^#^P<0.05 among Groups A, B, and C; *P<0.05 compared with Group A. The chi-squared test was used for statistical analyses.

### Laboratory findings among patients with KD with different ages

We then compared laboratory data, including white blood cell count, neutrophils, hemoglobin, platelet count, C-reactive protein levels, erythrocyte sedimentation rate, and the levels of procalcitonin, serum sodium, lactate dehydrogenase, alanine aminotransferase, aspartate aminotransferase, and brain natriuretic peptide, in patients with KD according to age. However, there were no significant differences in these laboratory findings among the different age groups (Table 3).

**Table 3:** Laboratory data of the different Kawasaki disease age groups.

Variable	Group A	Group B	Group C	F or χ^2^ value	P value
WBC (10^9^/L)	17.31±6.31	16.08±5.65	16.03±4.32	3.034	0.087
N (10^9^/L)	10.36±4.47	10.94±4.94	10.79±4.51	0.151	0.860
Hb (g/L)	106.28±12.72	103.75±10.89	109.29±7.39	0.860	0.401
PLT (10^9^/L)	395.24±141.33	357.08±111.38	376.00±78.25	0.710	0.503
CRP (mg/L)	90.42±58.25	103.80±66.85	112.44±60.13	0.654	0.521
ESR (mm/h)	66.44±25.34	63.37±26.63	65.35±25.07	0.588	0.611
PCT (μg/L)	10.38±43.96	4.93±40.44	1.23±2.30	0.310	0.734
Na (mmol/L)	138.00±2.41	137.30±3.07	136.18±3.24	1.867	0.158
LDH (U/L)	405.92±125.03	449.08±197.85	443.94±211.52	0.538	0.585
ALT [U/L]	28 (20-60)	38 (19-125)	27 (18-73)	5.287	0.071
AST [U/L]	38.5 (29-56)	41 (31-74)	31 (23-70)	4.455	0.108
BNP [U/L]	513 (269-1058)	332 (183-819)	308 (158-545)	4.319	0.115

Group A: <1 year; group B: ≥1 and <5 years; group C: ≥5 years. WBC: white blood cell; N: neutrophils; Hb: hemoglobin; PLT: platelet count; CRP: C-reactive protein; ESR: erythrocyte sedimentation rate; PCT: procalcitonin; Na: serum sodium; LDH: lactate dehydrogenase; ALT: alanine aminotransferase; AST aspartate aminotransferase; BNP: brain natriuretic peptide. Data are reported as means±SD or median with interquartile range. The chi-squared test, Fisher's (F) exact test, or one-way analysis of variance were used for statistical analyses.

### Echocardiographic findings among patients with KD with different ages

Initial echocardiographic findings indicated that there was no significant difference in the incidence of CALs among the different age groups. However, the incidence of CALs in group A was significantly higher than that in the other groups 2 months after diagnosis (P=0.036, Table 4). Other echocardiographic findings, including pericardial effusion (16 cases, 4.0%), decreased left ventricular function (9 cases, 2.3%), mitral regurgitation (6 cases, 1.5%), and aortic regurgitation (10 cases, 2.5%), were not significantly different among the age groups.

**Table 4:** Echocardiographic findings in the different Kawasaki disease age groups.

Variable	Group A	Group B	Group C	χ^2^ value	P value
Initial CAL, n (%)	15 (24.2%)	45 (15.7%)	8 (16.0%)	2.621	0.270
CAL at 2 months follow-up, n (%)	12 (19.4%)	24 (8.4%)*	5 (10.0%)*	6.633	0.036^#^
Pericardial effusion, n (%)	4 (6.5%)	9 (3.1%)	3 (6.0%)	2.557	0.293
Decreased left ventricular systolic function, n (%)	1 (1.6%)	6 (2.1%)	2 (4.0%)	1.127	0.651
Mitral regurgitation, n (%)	3 (4.8%)	3 (1%)	0 (0%)	4.226	0.097^#^
Aortic regurgitation, n (%)	2 (3.2%)	6 (2.1%)	2 (4%)	1.407	0.516

Group A: <1 year; group B: ≥1 and <5 years; group C: ≥5 years. CAL: coronary artery lesions. ^#^P<0.05 among Groups A, B, and C; *P<0.05 compared with Group A. The chi-squared test was used for statistical analyses.

## Discussion

In our study, the male/female ratio was 1.84, similar to those from several national epidemiological surveys on KD (9–11). However, there was no obvious difference in sex distribution among the different age groups.

Previous studies have suggested that KD patients with different ages have different manifestations of the disease (12,13). In our study, the prevalence of symptoms of oral changes, conjunctivitis, rash, and changes in the extremities was not significantly different among the different age groups. However, the prevalence of cervical lymphadenopathy was lower in patients aged <1 year than in those at other ages, and these patients were more likely to have incomplete clinical presentation. These results showed similarities with earlier results obtained by Cho et al. (14), who reported that the least common symptom in patients with KD aged <1 year was cervical lymphadenopathy. We also found that the duration of fever before admission was significantly longer in patients aged <1 year than in the other age groups, which presumably was attributed to the high proportion of incomplete KD in these patients.

Gastrointestinal disorders include many different clinical manifestations and echographic findings, which have been reported in approximately 20 to 35% of patients with KD (15–18). In a recent study, Fabiet al. (17) found that patients with KD and gastrointestinal involvement were significantly younger. Our findings were similar as gastrointestinal disorders occurred more frequently in patients aged <1 year than in those aged 1-5 years. Neurological involvement is a relatively unrecognized complication of KD, and it includes extreme irritability (19), aseptic meningitis (20), peripheral facial nerve palsy (21), and sensorineural hearing loss (22). However, neurological involvement has been reported mainly in case reports and is poorly characterized. In our cohort, the proportion of infants aged <1 year with neurological involvement was significantly higher than that of older patients.

Redness at the BCG inoculation site was described as a positive sign in the American Heart Association scientific statement (7). In a Japanese nationwide epidemiological survey, Uehara et al. (23) reported that 49.9% of patients with KD and a history of BCG vaccination had redness at the BCG inoculation site. Moreover, of those patients aged 3-20 months, more than 70% had redness at the BCG inoculation site. In this study, young patients were also more prone to developing redness at the BCG inoculation site. Furthermore, redness at the BCG inoculation site was more prevalent than cervical lymphadenopathy and changes in the extremities in patients with KD aged <1 year. Redness at the BCG inoculation site appears to be a useful criterion in the diagnosis of KD in younger infants.

Musculoskeletal involvement includes arthralgia and arthritis. Previous studies showed that the prevalence of arthritis in KD ranged from 7.5 to 12.7% (24), and older children with KD were more prone to developing arthritis. Similar results were found in our study, where the frequency of musculoskeletal involvement in patients aged <1 year was lower than that in older patients. We speculate that this finding was mainly due to the difficulty of diagnosing arthritis in this age group.

Respiratory symptoms are frequently observed in children with KD during the acute phase (25). Lee et al. (26) reported that respiratory symptoms were found in 31.9% of patients with KD, and there was no significant difference in age distribution between patients with KD and without respiratory symptoms. Genitourinary involvement of KD is uncommon and most patients with KD and genitourinary involvement have pyuria (7). Watanabe (27) reported that patients with KD and pyuria were more likely to be aged <1 year. In our study, no significant difference was found in the prevalence of respiratory involvement or genitourinary involvement among the different age groups.

Cardiovascular manifestations represent the major contributors to morbidity and mortality related to KD. Several studies have reported that young age is a risk factor for CALs (28,29). Fernandez-Cooke et al. (11) studied the epidemiology and clinical features in KD in Spain and found that children aged <12 months develop coronary aneurysms more frequently than older children. In this study, we found that the rate of CALs in the acute phase of KD in patients aged <1year was not significantly different than that in older patients. However, there was a significantly higher incidence of CALs in younger patients at 2 months after initial echocardiography compared with older patients, which was similar to previous studies.

There are several limitations in this study. First, there was a limited number of patients enrolled in groups A and C, and thus more studies are required to investigate the specific characteristics of these patients. Second, detection of symptoms before hospitalization was partly based on interviews with the parents, who may have failed to recognize some symptoms.

## Conclusions

Although other authors had a similar approach, our study added to the current knowledge on KD. Our study showed that cervical lymphadenopathy and musculoskeletal symptoms occurred more frequently in older patients than in younger patients. Gastrointestinal symptoms, neurological symptoms, and redness at the BCG inoculation site occurred more frequently in patients aged <1 year than in older patients, and these patients were more likely to develop CALs.
